# Association between the Composition of Drinking Water and Cognitive Function in the Elderly: A Systematic Review

**DOI:** 10.3390/ijerph21030362

**Published:** 2024-03-19

**Authors:** Annie Wasick, Yeonsoo Kim

**Affiliations:** Nutrition and Dietetics Program, Central Michigan University, 1200 South Franklin Street, Mt. Pleasant, MI 48859, USA; wasic1ae@cmich.edu

**Keywords:** trace elements, drinking water, cognitive function, elderly

## Abstract

The prevalence of dementia increases with nearly 10 million new cases each year, with Alzheimer’s disease contributing to 60–70% of cases. Environmental factors such as drinking water have been evaluated to determine if a relationship exists between trace elements in drinking water and the risk of developing cognitive disorders in the elderly. The purpose of the current systematic review was to evaluate an association between the composition of drinking water and cognitive function in the elderly. In accordance with the Preferred Reporting Items for Systematic Review and Meta-Analyses guidelines, a literature search was conducted using PubMed and CINAHL databases. A total of 10 studies were included in the current systematic review. Aluminum is the most commonly evaluated trace element in studies (*n* = 8), followed by silica (*n* = 5), calcium (*n* = 4), and fluoride (*n* = 4). Aluminum exposure showed an increased risk of cognitive decline in four studies, with no association reported in the other studies. Higher silica and pH levels were shown to be protective against a decline in cognitive function. A similar protective effect of calcium was found in two studies. Future research should measure multiple trace mineral levels in all water sources to evaluate the impact on cognitive function.

## 1. Introduction

According to the World Health Organization [WHO], in 2019, 55 million people lived with dementia worldwide, and the number is expected to continue to grow [1]. Dementia places the people living with it and their families in significant physical, emotional, and financial distress [1,2]. Dementia care costs also become a burden to society. According to the WHO, the estimated total global societal cost of dementia was 1.3 trillion dollars in 2019, and the cost is expected to more than double by 2030 as the rate of dementia and cost of care increase [1]. 

Dementia is a general term characterized by the loss of cognitive functions, including an impaired ability to remember, think, and reason [3]. Alzheimer’s disease (AD) is the most common form of dementia and is the seventh leading cause of death among all diseases [4,5]. AD is irreversible and leads to cognitive decline, alteration in memory, less ability to do daily activities, and decreased ability to care for oneself. It can change the personality of individuals due to the slowly destroyed brain caused by the disease [4]. 

The cause of AD is unknown, but genetic findings and environmental factors have been a focus of research. Among the environmental factors, air pollution and continuous exposure to fine solid and liquid particles in the air from fuel combustion and fires have been shown to increase the risk of cognitive decline [4,6]. Trace elements in drinking water are another environmental factor being studied in relation to cognitive decline [7,8,9,10,11,12,13,14,15]. 

Tap water may contain various levels of trace elements and supply the intake of these for populations. Water was the most commonly consumed nonalcoholic beverage among Americans aged 60 and over in 2015–2018 [16]. A 15-year follow-up of the Personnes âgées Quid (PAQUID) study showed that 95.9% of the aluminum was supplied by tap water [13]. Aluminum has been the focus of many studies to evaluate if the intake of this trace element from various sources has a toxic effect and increases the risk of developing AD [10,11]. Aluminum has been suggested to contribute to AD development after finding aluminum in neurofibrillary tangles in the brains of patients with AD [10,17,18]. 

Calcium has also been a focus for its potential protective role by decreasing the gastrointestinal absorption of aluminum [10]. Trace elements may also interact with each other and impact the absorption rates of other elements [7]. Drinking water may be supplied by deep drill holes, springs, wells, and lakes or rivers, and the trace element content of these sources may vary between regions [10,11,12,13]. Moreover, bottled water is another water source, and each brand has different trace mineral concentrations, which may impact the exposure level of individuals as well [9,13]. 

Due to multiple trace elements being supplied by drinking water, determining if any of these elements contribute to the development of cognitive disorders is important. Some trace elements may be protective against cognitive disorders, while others may be harmful. It is important to determine if a relationship exists to reduce the risk of developing cognitive disorders, improve the lives of individuals at risk, and reduce the impact on society. The current systematic review aims to evaluate if a relationship exists between the composition of drinking water and cognitive function in the elderly.

## 2. Materials and Methods

### 2.1. Protocol

The present systematic review on the trace element content of drinking water and cognitive function was conducted in accordance with the general principles of Preferred Reporting Items for Systematic Reviews and Meta-Analysis (PRISMA) 2020 [19].

### 2.2. Search Strategy

A literature search was conducted on the electronic databases PubMed and Cumulative Index to Nursing and Allied Health Literature (CINAHL). A search of relevant articles from the reference list of included full-text articles was also conducted. Keywords used in the literature search included combinations of the following: “aged”, “elderly”, “drinking water”, “tap water consumption”, “trace elements”, “cognition”, “cognitive impairment”, “Alzheimer disease”, and “dementia”. All articles were imported into Rayyan, and duplicates were removed using the “find duplicate” function. Titles and abstracts were screened to determine if the articles fit the eligibility criteria, and those that did not meet them were excluded. Once the titles and abstracts met all inclusion criteria, full texts were reviewed.

### 2.3. Eligibility Criteria

To be included in the current systematic review, studies should meet the following criteria: adults aged 65 years or older, trace elements in drinking water measured, cognitive function evaluated by validated instruments, cohort or cross-sectional studies, published between 1990 and 2022, and available in English. Articles were excluded if the participants were below the age of 65 years, did not evaluate trace element content from drinking water sources, only evaluated hydration or dehydration impacts and not water composition, or did not have a validated cognitive function evaluation test. 

### 2.4. Data Extraction and Quality Assessment

The present systematic review was written by an independent author and assessed by one author. The studies included in the review were interpreted by the two authors. Data extracted from the studies were analyzed by two authors and included in the review. Studies were assessed based on selection, performance, measurement, and bias. The study Quality Assessment Tool developed by the National Heart, Lung, and Blood Institute (NHLBI) was used [20]. The quality assessment of the studies is shown in Table 1. 

## 3. Results

### 3.1. Search Results

**Figure 1:**
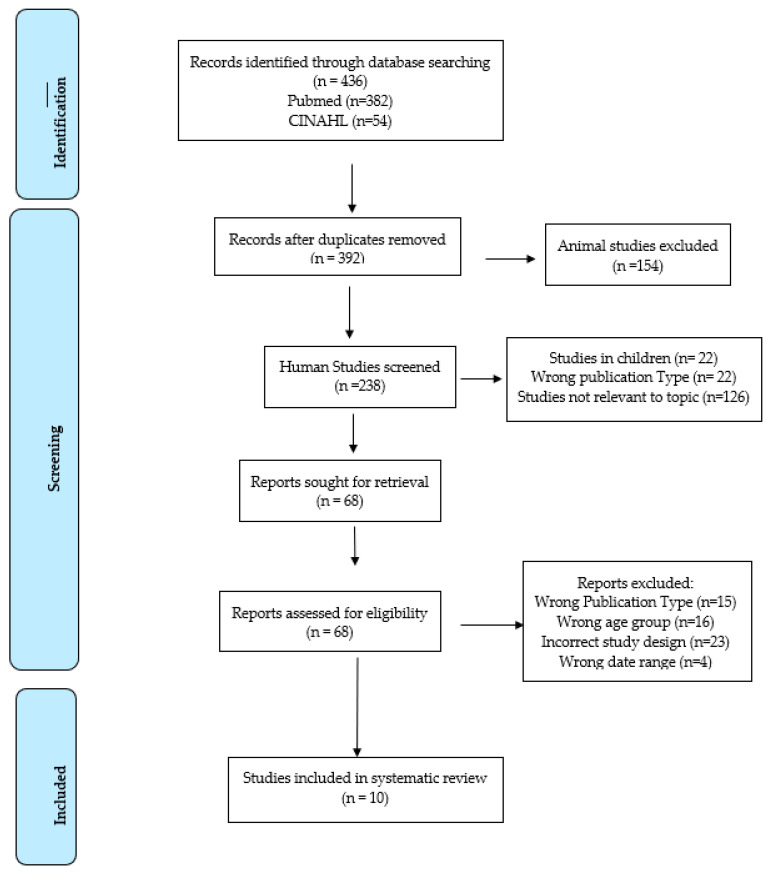
PRISMA flow diagram.

A total of 436 articles were found within the search parameters and imported into Rayyan. Duplicates, which totaled 44, were removed using the duplicate identification function. After reviewing full texts, 10 articles met the inclusion criteria and were included in the current systematic review (Figure 1). 

### 3.2. Characteristics of Included Studies

A total of 22,141 elderly people aged 65 years and older were included in the current systematic review. Of the 10 studies included, 7 were conducted in European countries [9,10,11,12,13,21], 2 in Canada [8,14], and 1 in China [22]. The trace elements evaluated in each study are as follows: aluminum in eight studies [8,9,10,11,12,13,14,15]; silica in five studies [9,11,12,13,14]; calcium in four studies [7,9,10,11]; fluoride in four studies [7,10,11,15]; each cadmium, iron, lead, selenium, and zinc in one study [7]. 

### 3.3. Cognitive Function Tests

Multiple validated methods were used to evaluate cognitive function: the Mini-Mental State Exam (MMSE) [10,11,13,14,15,21], the Modified Mini-Mental State (3MS) screening test [8], the Short Portable Mental Status Questionnaire (SPMSQ) [9], and the Community Screening Interview for Dementia (CSID), but slightly modified for Chinese translation [7]. In addition, a 1-h interview with a psychometric evaluation was conducted by a trained psychologist to evaluate for impairment of other cognitive functions and interference with social or professional life, according to DDM-III-R criteria [12]. Furthermore, multiple cognitive function tests were used in one study. The tests included the MMSE, the Italian version of the Story Recall Test, The Raven’s Colored Progressive Matrices (CPM) test, The Trail Making test (TMT), The Digit Span Digit Symbol from the Wechsler Adult Intelligence Scale, Five subtasks from the Luria Nebraska Neuropsychological Battery, Finger Tapping test and the Simple Visual Reaction Time from the computerized Swedish Performance Evaluating System battery, Catsys Tremor 7.0 by Danish Product Development, and Sniffin Sticks-Olfactory Screening test [21]. 

### 3.4. Results of Trace Minerals in Drinking Water and Cognitive Function

The results of the studies included in the current systematic review are summarized in Table 2. A Canadian cohort study with 1924 elderly aged 70 years or older who had lived in the residence for an average of 44 years was conducted in the region of Saguenay-LacSaint-Jean, Quebec, in 1995–1996 to determine the relationship between long-term exposure to various aluminum forms and AD development. Concentrations of total aluminum, total dissolved aluminum, organic monomeric aluminum, polymeric aluminum, and aluminum hydroxide were measured in drinking water collected from 54 municipalities in the region. Fifty-four drinking water supplies included 14 river water, 17 lake water, 19 groundwater, and 4 completely treated water from a water purification plant. Surface water (river and lake water) accounted for approximately two-thirds of the drinking water supplies in this study. Total dissolved aluminum levels in surface water were higher than in groundwater (2.51, 1.50, and 0.33 µM from rivers, lakes, and groundwater, respectively). In particular, polymeric and monomeric aluminum were dominantly present in the river and lake water, while monomeric aluminum and aluminum hydroxide were high in the groundwater. No association was found between AD development and exposure to aluminum in any of the forms except for organic monomeric aluminum after the data were adjusted for genetic characteristics, such as the presence of the ApoE-e4 allele and cases of dementia or AD among first-degree relatives (odds ratio (OR) 2.67; 95% confidence interval (CI) 1.04–6.90, *p* = 0.01) [8]. 

A Canadian national longitudinal study, The Canadian Study of Health and Aging, was conducted in 35 Canadian municipalities from 1991–1992 to 2001–2002. The participants were required to have residential history data for at least seven years at baseline, and their average age was 75.5 years. Based on tap water, water trace element concentrations, pH, water treatment, or distribution facilities in 35 municipalities were collected. In addition, a questionnaire was used to obtain data on consumption of wine, coffee, or tea. The average trace element concentrations in drinking water were reported as follows: 134.07 ± 152.47 μg/L for aluminum; a pH of 7.4 ± 0.8; 0.55 ± 0.45 mg/L for fluoride; 2.69 ± 2.04 mg/L for silica; and 48.9 ± 48.6 μg/L for iron. No associations were found between AD development and aluminum, pH, fluoride, silica, and iron in drinking water [14]. 

As the first cohort study in European countries in dementia-related research, the PAQUID study is a prospective cohort study established in France in 1988 to evaluate social environments, genetic conditions, cognitive function, and depressive symptoms. The population represents the elderly aged 65 years and older living at home in the 75 parishes of Gironde and Dordogne in southwestern France. The participants stayed in the same parishes for 41 years on average. At baseline, information on the water supplies of 78 areas of drinking water for the past 10 years was obtained by the sanitary administration. More than half (58%) of the water supplies were deep drill holes, and 30% were springs, followed by 7% wells and 5% lakes or rivers. Finally, the measurements of the water supplies in 72 areas were conducted after six areas were excluded due to measurements not being collected [10,11]. The PAQUID study was conducted with 3777 French elderly people with a mean age of 75.2 years at baseline found that the prevalence of cognitive impairment was impacted by the calcium and aluminum content of drinking water, depending on pH levels. The association between cognitive impairment and aluminum exposure was positive up to a pH of 7.3 (95% CI 6.3–7.78) and negative beyond that level. However, calcium concentrations in drinking water of 75 mg or higher showed a decreased risk of cognitive impairment (OR 0.76, 95% CI 0.61–0.95, *p* = 0.01). In this study, the following mean concentrations of trace elements were reported: aluminum of 33 μg/L ranging from 0 to 940 μg/L; calcium of 72.6 mg/L ranging from 8.6 to 148 mg/L; fluorine of 0.28 mg/L ranging from 0.03 to 2.03 mg/L; pH of 7.55 ranging from 6.86 to 8.54. In a subsample of 2404 participants, approximately 38% of the participants used aluminum cooking utensils, and only 4.9% were identified as exclusive users. There was no correlation between cognitive impairment and the use of aluminum cooking utensils. In the same subsample, 58.4% drank plain water, 77.9% drank tea or coffee daily, and 47% consumed both bottled water and tap water [10].

Secondary data analysis of the PAQUID study at baseline was conducted to investigate the protective effect of silica in water on cognitive function and to measure the association between cognitive impairment and other trace minerals, including magnesium, fluorine, zinc, iron, and copper. When silica and pH were low in drinking water, exposure to an aluminum concentration of 3.5 μg/L appeared more likely to cause cognitive impairment than those not exposed to aluminum (OR 3.94, 95% CI 1.39–11.2). In regions where high aluminum, low silica, and low pH contents were present in drinking water, the risk of cognitive impairment was more significant than in drinking water containing low aluminum, high silica, and high pH contents. High aluminum, high silica, and high pH had a 25% lower risk of cognitive impairment than low aluminum, high silica, and high pH in a larger number of regions. This indicates a possible protective effect of silica when aluminum levels are high. No association was found between the risk of cognitive impairment and magnesium, iron, zinc, and copper concentrations in drinking water [11]. 

An eight-year follow-up of the PAQUID study with 2698 subjects aged 65 years or older was conducted to assess incident cases of dementia or AD. The results of the chemical analyses of drinking water made by the sanitary administration between 1991 and 1994 were collected. The mean of all the drinking water components was then computed, weighing the time period of use of each water supply over the past 10 years and the relative contribution of each water supply. The elderly exposed to an aluminum concentration greater than 0.1 mg/L had a 2.03 times higher risk of developing dementia than the individuals living in areas with lower aluminum concentrations (95% CI = 1.23–3.34, *p* = 0.006). An inverse relationship was found with the risk of developing AD or dementia when silica concentrations were greater than or equal to 11.25 mg/L (relative risk (RR) = 0.75, 95% CI = 0.58–0.96, *p* = 0.023). Mineral water consumption was collected from 1638 participants without AD at the 3-year follow-up. In the participants, 48% drank mineral water daily, and 105 elderly developed dementia between the 3-year and the 8-year follow-up. After adjusting educational level, wine intake, residence place, and silica levels, this subsample showed a high risk of developing dementia for aluminum ≥0.1 mg/L (RR 2.89, 95% CI 1.51–5.52, *p* < 0.001). Given that aluminum cooking utensils could supply significant amounts of aluminum, statistical analysis including the use of aluminum cookware as a variable was conducted. However, the use of aluminum cooking utensils was not associated with the risk of dementia (HR 1.04, *p* = 0.86) [12]. 

Data from another study were available from the combined 15-year follow-up of the PAQUID cohort and the Aluminium Maladie d’Alzheimer cohort study, with a total of 1925 participants with a mean age of 82.4 years. The chemical analysis results of drinking water provided by the sanitary administration between 1991 and 1994 were used to compute the mean of all measures of aluminum and silica. Additionally, the history of the water distribution network over the previous ten years was evaluated to measure the participants’ past exposure. This study included specific questions regarding the daily intake of tap water, including tap water used in making tea, coffee, soup, or alcoholic drinks, and the intake of bottled water and the brand most frequently consumed. The average intake of drinking water was 0.94 L/day. Tap water and bottled water were the only sources of water intake for 43.7% and 40.3% of the participants, respectively. The daily intake of aluminum from drinking water was 0.025 mg, ranging from 0 to 1.03 mg. Most aluminum (95.9%) was supplied by tap water, while only 4.1% was provided by bottled water. The daily intake of silica was 13.37 mg, with a minimum of 0 and a maximum of 108 mg. Unlike aluminum, more than half of the silica (59%) was supplied by bottled water. A daily intake of aluminum greater than or equal to 0.1 mg from drinking water increased the risk of dementia (adjusted RR 2.26, 95% CI 1.00–5.07, *p* = 0.049), while silica intake of 10 mg/day was associated with a reduced risk of dementia (adjusted RR 0.89, 95% CI 0.81–0.99, *p* = 0.036). However, geographic exposure to aluminum or silica from tap water was not related to dementia development [13]. 

In a study conducted in rural China (Henana and Shandong provinces of northern China) with elderly Chinese with a mean age of 72.1 years old who had resided in the area over the past 40–50 years, 20 water sources within the villages in the provinces were identified, and trace elements and pH levels in the sources were measured. The following are the mean concentrations of trace elements reported: cadmium of 0.22 μg/L, ranging from 0.07 to 0.49; calcium of 72.2 mg/L, ranging from 30.2 to 153.10; fluoride of 2.6 mg/L, ranging from 1.45 to 4.70; iron of 266.7 μg/L, ranging from 15.4 to 1330; lead of 2.2 μg/L, ranging from 0.29 to 9.46; selenium of 0.53 μg/L, ranging from 0.05 to 4.24; zinc of 15.21 μg/L, ranging from 0.15 to 236; and pH of 7.4, ranging from 7.1 to 7.8. Calcium had a negative quadratic effect on cognitive function after adjusting for age, sex, and education (r = −0.0009, *p* < 0.01), meaning that calcium was protective against a decline in cognitive function up to 86 mg/L in drinking water. In contrast, lead had a positive quadratic effect (r = 0.072, *p* = 0.03); as the lead level increased up to a point, cognitive function decreased. However, as the lead level continued to increase, cognitive function also increased. Both fluoride and iron had positive linear associations with cognitive function (r = 0.899, *p* = 0.02; r = 0.0039, *p* = 0.01, respectively), while selenium and zinc showed negative linear relations (r = −0.656, *p* < 0.01; r = −0.13, *p* = 0.01, respectively). No relationship between fluoride, iron, or lead and the risk of cognitive decline was found after adjustment for the other trace elements [7]. 

A study was conducted in Brescia, Italy, the Valcamonica, to evaluate manganese exposure through environmental factors of air, soil, and tap water with 255 elderly participants aged 65–75 years who had locally resided since at least the 1970s. Inhalation exposure to airborne particles was measured using 24-h personal air monitoring, and tap water was sampled from each participant’s primary residence. Average airborne manganese exposure was 26.41 n/gm^3^ in Valcamonica and 20.96 ng/m^3^ in the reference area of Garda Lake. The average manganese content of the soil was 1026 ppm in Valcomonica and 421 ppm in the reference area of Garda Lake. The average manganese content of drinking water in Valcomonica was below 1 μg/L. Exposure to manganese from soil and air showed associations in multiple cognitive function tests, including the odor identification score measured by the Sniffin Stick test, The Raven’s Colored Progressive Matrices, and the Trail Making test. Manganese in drinking water was below the lower limit and was not associated with cognitive function [21]. 

A study conducted in Zurich evaluated 800 participants aged 81 to 85 who lived for more than 15 years in nursing homes located in areas with low aluminum levels in drinking water compared to nursing homes with higher aluminum concentrations in drinking water. The aluminum levels in drinking water in the city of Zurich have remained constant in each district since 1974, varying from district to district. The district with low aluminum levels in the drinking water is mainly supplied with ground water that does not need any treatment, and the aluminum concentration in the drinking water was less than 10 µg/L. In contrast, the district with a higher level of aluminum in the drinking water is mainly supplied with lake water from a water treatment plant undergoing aluminum sulfate treatment. The mean aluminum concentration in drinking water was approximately 100 µg/L, which was obtained from the water treatment plant. The mean serum aluminum level for participants in the low aluminum content area was higher than that for those living in the high aluminum level areas (4.2 ± 3.1 vs. 1.7 ± 2.4 μg/L, *p* = 0.035). There was no difference in urinary aluminum levels between participants in the two areas. The mean urinary aluminum to creatinine ratio was higher in the elderly in the lower aluminum area than those in the higher aluminum area (624 ± 1.179 vs. 130 ± 520, *p* = 0.0004). No difference was found in MMS test results between the two areas, suggesting no contribution of aluminum content in drinking water to dementia development [15]. 

The Epidemiology of Osteoporosis (EPIDOS) study conducted in 1992–1994 was a cross-sectional study of women aged 75 years or greater living in five geographic areas of France (Amiens, Lyon, Montpellier, Paris, and Toulouse). In a follow-up to the cohort in Toulouse in 1999–2000, cognitive function was measured using the SPMSQ, MMSE, and Grober and Buschke tests. During the first five-year follow-up, 72 women were diagnosed with AD. At the 7-year follow-up, 450 women were normal, 58 had mild cognitive impairment, 38 had AD, and 96 had another type of dementia. A questionnaire was used to measure the daily intake of tap water, including water used in making tea or coffee, mineral water, and the brand of mineral water most frequently consumed. In addition, water composition data for the tap water in each city was obtained from the local water companies. The average daily water consumption was 0.94 L. Tap water was the sole source of water consumption for 48.1% of the participants, while 31.3% of the women drank only mineral water. The daily intake of trace elements supplied by drinking water is as follows; aluminum 0.0231 ± 0.025 mg/day; silica 10.17 ± 10.01 mg/day; and calcium 134.8 ± 154.1 mg/day. Only 5.6% of aluminum was supplied by mineral water, whereas 72.2% silica and 69.1% calcium were provided by mineral water. Women with a daily silica intake of less than 4 mg showed a 2.72 times higher risk of developing AD than women with a silica intake of greater than 12 mg per day (95% CI 1.09–6.86, *p* = 0.0316). No evidence was found between aluminum and calcium and the risk of developing AD [9]. 

## 4. Discussion

The present systematic review found a relationship between pH, aluminum, calcium, and silica intakes from drinking water and the development of cognitive disorders such as AD. Long-term aluminum exposure increased the risk of cognitive disorder development after multiple confounding factors were adjusted [8,10,11,12,13]. Silica was found to have a protective effect in four studies [9,11,12,13] with one study finding that if silica intake was less than 4 mg per day, the risk of AD development increased [9]. Two studies found a protective effect of calcium [7,10]. No relationship was found with other trace elements.

Aluminum is the most abundant element and is used as a coagulant to reduce organic materials in water, causing a reduction of organic materials, color, turbidity, and microorganism levels. Aluminum levels depend on water quality control and operational factors at water treatment facilities. Foods with aluminum-containing additives are the major contributor, although the aluminum present in water has higher biological activity than that in other sources [23]. Aluminum may bind to phosphate groups of DNA and RNA and impact the expression of genes that are essential for brain function [18]. Aluminum is not essential for life and is a neurotoxin that may lead to cognitive disorders, including AD [18,24,25]. Aluminum maltolate has also been shown to be involved in cytokine and neurotrophic balance and increased neurodegeneration. It has also been shown to decrease the expression of nerve growth factor and brain-derived neurotrophic factor, which impacts gene expression and can increase neurodegeneration [17]. 

In addition to the total aluminum intake, pH may affect aluminum bioavailability. Citrate enhances aluminum absorption [26], and a higher prevalence of dementia was reported in England, where drinking tea with lemon frequently occurs [27]. Moreover, acid rain [28] and nitrogen fertilizers [29] make soil and water acidic, accelerating the dissolution and mobilization of high aluminum levels into water. 

Silica may protect against AD development by reducing the transport of toxic substances to the brain. Silica may reduce the absorption of aluminum and therefore reduce the risk of developing AD [30,31]. Calcium has been found to have a protective role in AD development, while oversupplementation of calcium has also been shown to increase the risk of AD. Extracellular accumulation of amyloid plaques and intracellular neurofibrillary tangles in the brain is present in AD, and calcium dysregulation has been shown to induce synaptic deficits and promote the accumulation of the plaques and neurofibrillary tangles [32,33,34,35]. 

The rural Chinese cohort study compared the National Sanitary Standards for Drinking Water in China with eight trace element levels and pH in 20 water sources measured in rural northern China. Seven trace element levels met the National Sanitary Standards. However, iron content in the water supply ranged from 15.4 to 1330 µg/L, although the Chinese limit for iron is 300 μg/L. This indicates that the population residing in some rural areas of northern China may drink water with much higher iron concentrations than the reference [7]. Chronically elevated ingestion of iron may lead to increased oxidative stress and inflammation, which can increase the risk of chronic diseases such as cancer, heart disease, neurological diseases, and aging [36]. 

Different countries and regions may allow for higher limits on certain trace elements, which could impact exposure levels for individuals. It is important to evaluate the relationship between the content of trace elements in water and the risk of cognitive disorder development to identify if any regulations need to be adjusted to reduce the risk of future harm. Water supply systems may need to change, or water may need to be treated to reduce the trace element content. 

Some strengths of the studies included large sample sizes for participants ranging from 255 [21] to 4507 subjects [14]. All the studies also used validated cognitive assessment tools to measure cognitive function/impairment, allowing cognitive functions to be assessed accurately. Collecting and adjusting confounding factors such as education level, smoking status, primary occupation, and physical activity level resulted in accurate correlations to trace elements increasing cognitive decline. Studies suggest that lower educational levels and financial status may contribute to a greater risk of AD development [4,37,38]. Individuals with lower educational levels may have less mentally stimulating jobs and a lower socioeconomic status, which leads to poor nutrition and less access to health care. People with lower socioeconomic status are more at risk for smoking and developing diabetes and cardiovascular disease, which all may increase the risk of cognitive disorders [4]. Increased physical activity, such as walking, has been shown to reduce the risk of cognitive decline [22,39,40,41]. Tobacco use also places individuals at an increased risk of vascular disease and increases the risk for AD development [42]. Occupations primarily outdoors, focused on using substances for cleaning and manufacturing, or involving routine use of pesticides place participants at an increased exposure level to trace elements and contribute to the development of cognitive disorders [8,14,43,44,45]. These factors may place participants at a higher risk than others in the population and should be considered to accurately evaluate the risk of cognitive disorders from trace elements in the water. Moreover, prospective studies allowed a longer time frame of exposure to trace elements and evaluated the risk of cognitive decline over a more significant period [9,12,13,14]. Trace elements may build up over time, and the risk of cognitive decline may take years, so more time for follow-up studies allows for more accurate data. All the studies evaluated trace elements in water composition multiple times over the years. It resulted in more precise exposure scores to be calculated, considering that water makeup may have variations during different times of the year [8,14]. The PAQUID studies collected water samples from various sources, including deep drill holes, springs, wells, lakes, and rivers, during different seasons of the year that contributed to the main water supply [7,10,11,12,13]. 

Some limitations may include that the majority of studies included in the current systematic review did not collect the water intake from various sources, such as spring or mineral bottled water, water used to make tea or coffee, and food sources [7,8,14,15,21]. Therefore, the estimation of trace minerals mainly based on tap water would underestimate the total intake of trace minerals. Participants can be exposed to trace elements through soil, diet, and air as well [7]. Crops may be grown in areas where the soil has a higher content of certain trace elements, possibly contributing to the ingestion of higher amounts of certain trace elements [7,15,21]. Participants may also take dietary supplements or have diets where items higher in certain trace elements are more regularly consumed based on the region’s food supply [21]. Participants may also cook with tap water, and the full amount of exposure to the trace elements may not have been accounted for, but the process of cooking may also alter the exposure rate [14]. Certain cohort studies may not have considered that certain members of the population may have moved from other regions or passed away, but two studies had inclusion criteria of at least seven years and at least 15 years of living in the region [14,15]. Another was conducted in a village in rural China, and this population likely had an increased rate of residing in the area at longer rates [7]. 

Future research could consider the intake of trace minerals from various water sources, diets, medications, etc. The 2005–2006, 2007–2008, and 2009–2019 National Health and Nutrition Examination Survey (NHANES) reported that on average, 56% of drinking water volume comes from tap water, while the other 44% comes from bottled water [46]. Only one study measured aluminum, silica, and calcium concentrations in mineral waters [9]. The mineral water Evian had an undetectable level of aluminum, 15.2 mg/L of silica, and 82 mg/L of calcium [15]. Due to the significant content of silica and calcium in mineral water, it is important to measure trace mineral concentrations in all water sources. Moreover, it could consider the intake of other water sources for people who have moved from other regions. It may not be easy to conduct, but it would allow for more accurate information about lifetime exposure to certain elements. 

## 5. Conclusions

Determining if a relationship exists between tap water content and cognitive disorder development is important due to the significant impact cognitive disorders have on individuals and society. From the studies evaluated, two studies found a protective effect of calcium [7,10]. Silica and pH were shown to have a protective effect in certain concentrations as well [9,10,11,12,13]. Aluminum exposure was found to increase the risk of cognitive decline in two studies [8,10,11,12,13]. Factors such as educational level, occupation, and lifestyle factors may also impact the risk of developing dementia and AD and need to be adjusted for data analysis to accurately determine the role drinking water plays in the development of dementia and AD. Future studies to further evaluate the impact of trace elements from various sources, such as diet, beverages, and medication, especially aluminum and silica, will be beneficial to make a favorable impact on potentially reducing the prevalence of cognitive disorders. Moreover, multiple trace mineral interactions at various pH levels need to be investigated to prevent dementia and/or AD.

## Figures and Tables

**Table 1:** Quality assessment of the included studies.

Reference	Study Design	Possible Conflict of Interest Reported	Total Quality Score	Maximum Quality Score	Criteria Not Met
Gauthier et al., 2000 [8]	Cohort	No	12	13	Was a sample size justification, a power description, or variance and effect estimates provided? Not provided
Van Dyke et al., 2019 [14]	Cohort	No	12	13	Was a sample size justification, a power description, or variance and effect estimates provided? Not provided
Jacquim et al., 1994 [10]	Cohort	No	12	13	Was a sample size justification, a power description, or variance and effect estimates provided? Not provided
Jacquim-Gadda et al., 1996 [11]	Cohort	No	12	13	Was a sample size justification, a power description, or variance and effect estimates provided? Not provided
Rondeau et al., 2000 [12]	Cohort	No	12	13	Was a sample size justification, a power description, or variance and effect estimates provided? Not provided
Rondeau et al., 2009 [13]	Cohort	No	12	13	Was a sample size justification, a power description, or variance and effect estimates provided? Not provided
Emsley et al., 2000 [7]	Cohort	No	11	13	Was the exposure(s) assessed more than once over time? No Was a sample size justification, a power description, or variance and effect estimates provided? No
Lucchini et al., 2014 [21]	Cohort	No	12	13	Was a sample size justification, a power description, or variance and effect estimates provided? Not provided
Wettstein et al., 1991 [15]	Cross- sectional	No	14	14	All criteria were met
Gillette-Guyonnet et al., 2005 [9]	Cohort	No	11	13	Was the loss to follow-up after baseline 20% or less? No Was a sample size justification, a power description, or variance and effect estimates provided? No

**Table 2:** Summary of studies and outcomes.

Reference	Sample Size	Mean Age (yrs)	Region	Study Design	Trace Elements Measured	Cognitive Test Used	Results
Gauthier et al., 2000 [8]	1924, who lived in the residence for an average of 44 years	≥70	Saguenay-Lac-Saint-Jean Region, Quebec, Canada	Cohort	Aluminum in drinking water for 54 municipalities (14 river water, 17 lake water, 19 groundwater, and 4 completely treated water from a water purification plant)	3MS	- Mean total dissolved aluminum (mean ± SD, µM): 2.51 ± 1.92 in river water; 1.50 ± 1.38 in lake water; 0.33 ± 0.30 in groundwater; and 1.36 ± 1.28 in treated water. - Polymeric and monomeric aluminum were dominantly present in the river and lake water (47.02% and 35.97% for river lake; 44.34% and 36.5% for lake water), but monomeric aluminum and aluminum hydroxide were high in groundwater (54% and 31.1%). Here, 58% polymeric, 22.6% monomeric aluminum, and 18.9% aluminum hydroxide were composed of the treated water. - A positive association was reported between aluminum–molybdenum and cognitive impairment after adjusting multiple confounding factors (OR 2.67; 95% CI 1.04–6.90, *p* = 0.01). - No association was seen between AD and exposure to aluminum in any of the forms except aluminum–molybdenum.
Van Dyke et al., 2021 [14]	4507, who lived in the residence for at least 7 years at baseline	75.5	Saguenay-Lac-Saint-Jean Region, Quebec, Canada	Cohort, Canadian Study of Health and Aging (CSHA)	pH, aluminum, fluoride, silica, and iron in 35 municipal tap water	3MS	- All water parameters are as follows (mean ± SD): 134.07 ± 152.47 µg/L for aluminum; pH of 7.4 ± 0.8; 0.55 ± 0.45 mg/L for fluoride; 2.69 ± 2.04 mg/L for silica; 48.9 ± 48.6 μg/L for iron - No relationship was found between AD development and aluminum, pH, fluoride, silica, and iron.
Jacquim et al., 1994 [10]	3777, who lived in the residence for an average of 41 years at baseline	75.2	Gironde and Dordogne in southwestern France	Cohort, PAQUID study	Aluminum, calcium, and fluorine, pH in 72 water supplies (deep drill holes, springs, wells, and lakes or rivers)	MMSE	- All water parameters are as follows (mean ± SD): 33 ± 116.0 μg/L for aluminum; pH of 7.55 ± 0.29; 0.28 ± 0.42 mg/L for fluoride; 72.6 ± 34.5 mg/L for calcium. - Aluminum exposure was dependent on pH; it had a positive effect on cognitive impairment up to a pH of 7.3 (95% CI 6.3–7.78). - Calcium concentration in drinking water 75 mg or greater decreased the risk of cognitive impairment (OR 0.76, 95% CI 0.61–0.95, *p* = 0.01).
Jacquim-Gadda et al., 1996 [11]	3777, who lived in the residence for an average of 41 years at baseline	75.2	Gironade and Dordogone in southwestern France	Cohort, PAQUID study	Aluminum, calcium, pH, silica, magnesium, iron, zinc, copper, and fluoride in 72 water supplies (deep drill holes, springs, wells, and lakes or rivers)	MMSE	- All water parameters are as follows (min~max): 1.0~459 μg/L for aluminum; pH of 6.31~8.44; 0.05~1.83 mg/L for fluoride; 4.2~22.4 mg/L for silica; 0~0.71 μg/L for iron; 8.9~146.2 mg/L for calcium; 1.1~34.0 mg/L for magnesium; 0~0.17 mg/L for copper; 0~0.6 mg/L for zinc. - No relationship was reported between cognitive impairment and calcium. - When silica and pH were low in drinking water, exposure to an aluminum concentration of 3.5 μg/L in drinking water increased the risk of cognitive impairment compared to those not exposed to aluminum (OR 3.94, 95% CI 1.39–11.2). - High aluminum, low silica, and low pH levels increased cognitive impairment compared to low aluminum, high silica, and high pH contents in drinking water. - Silica had a protective effect when aluminum levels were high. - No association was found between cognitive function and magnesium, iron, zinc, and copper.
Rondeau et al., 2000 [12]	2698, who lived in the residence for an average of 41 years at baseline	≥65	Gironde and Dordogne in southwestern France	Cohort, 8 yr of follow-up PAQUID study	Aluminum, silica in 70 water supplies, and mineral water intake	MMSE	- Aluminum and silica levels in water supplies ranged from 0.001 to 0.459 mg/L and from 4.2 to 22.4 mg/L, respectively. - Subjects exposed to an aluminum concentration greater than 0.1 mg/L had a greater relative risk of dementia (OR 2.03, 95% CI 1.23–3.34, *p* = 0.006). - A negative relationship was seen with the risk of AD or dementia development when silica concentrations were greater than or equal to 11.25 mg/L (RR 0.75, 95% CI 0.58–0.96, *p* = 0.023). - There was no association between aluminum cooking utensil use and the dementia risk (HR 1.04, *p* = 0.86).
Rondeau et al., 2009 [13]	1925, who lived in the residence for an average of 41 years at baseline	82.4	Gironde or Dordogne in southwestern France	Cohort, 15 yr of follow-up PAQUID study and Aluminium Maladie d’Alzheimer study	Aluminum, silica in tap water, and bottled water	MMSE	- The mean intake of drinking water was 0.94 L/day. - The mean intake of aluminum and silica by drinking water was 0.025 ± 0.08 mg and 13.37 ± 10.76 mg, respectively. - A higher daily intake of aluminum ≥ 0.1 mg/day from drinking water increased the risk of dementia (adjusted RR 2.26, 95% CI 1.00–5.07, *p* = 0.049). - Silica intake of 10 mg/day was associated with a reduced risk of dementia (adjusted RR 0.89, 95% CI 0.81–0.99, *p* = 0.036).
Emsley et al., 2000 [7]	444 M; 572 F, who had lived in the same village over the past 40–50 years.	72.1 ± 5.1 M; 72 ± 5.5 F	Henan and Shandong provinces of northern China	Cohort	Cadmium, calcium, fluoride, iron, selenium, and zinc are found in 20 water sources within the villages	Chinese translation of Community Screening Interview for Dementia	- All water parameters are as follows (mean ± SD): 0.22 ± 0.112 μg/L for cadmium; pH of 7.4 ± 0.2; 2.6 ± 0.94 mg/L for fluoride; 72.2 ± 33.27 mg/L for calcium; 266.7 ± 307.8 μg/L for iron; 0.53 ± 0.946 μg/L for selenium; 3.6 ± 5.63 μg/L for zinc. - Calcium was protective against a decline in cognitive function up to 86 mg/L in drinking water. - Cognitive function reduced up to a point as the lead level increased. - A positive linear association was reported between cognitive function and both fluoride and iron levels. - No relationship was found between the risk of cognitive decline and selenium, fluoride, iron, or lead after adjustment for the other trace elements.
Lucchini et al., 2014 [21]	255, locally residing since at least the 1970s.	65–75	Valcomonia, Italy	Cohort, part of PHIME (Public Health Impact of Mixed Element Exposure)	Manganese in tap water	MMSE; The Italian version of the Story Recall Test; CPM; TMT; The Digit Span; Five subtasks from the Luria Nebraska Neuropsychological Battery; Finger Tapping test from the computerized SPES battery; Digit Symbol; Simple Visual Reaction Time from the SPES; Catsys Tremor 7.0 by Danish Product Development; Sniffin Sticks-Olfactory Screening test	- Exposure to soil and air manganese showed associations in multiple cognitive function tests, including the odor identification score measured by the Sniffin Stick test, The Raven’s Colored Progressive Matrices, and the Trail Making test. - Manganese in tap water was below the lower limit (1 μg/L) - No associations were found between tap water manganese content and its impact on cognitive function.
Wettstein et al., 1991 [15]	800, living for more than 15 years	81–85	Zurich- 2 city districts with a socioeconomically similar population and resident mean	Cross-sectional	Aluminum in the water supply	MMSE	- Areas with low aluminum levels (<10 µg/L) vs. areas with high aluminum levels (approximately 100 µg/L) - No significant difference was reported in the MMSE results between the elderly living in nursing homes located in areas with low aluminum levels in drinking water compared to those residing in nursing homes with higher aluminum concentrations in drinking water
Gillette-Guyonnet et al., 2005 [9]	1462	≥75	5 geographic areas of France (Amiens, Lyon, Montpellier, Paris, and Toulouse)	Cohort, EPIDOS study follow-up	Silica, calcium, and aluminum in city water supply and mineral water	SPMSQ; MMSE; Grober and Buschke test	- The daily intake of aluminum, silica, and calcium by drinking water is as follows (mean ± SD): 0.0231 ± 0.025 mg/day, 10.17 ± 10.01 mg/day, and 134.8 ± 154.1 mg/day, respectively. - Silica intake < 4 mg per day showed an increased risk of developing AD (OR 2.72, 95% CI 1.09–6.86, *p* = 0.0316). - No evidence was found between aluminum and calcium and the risk of developing AD.

3MS, Modified Mini-Mental State Examination; AD, Alzheimer’s disease; CI, confidence interval; CPM, Colored Progressive Matrices; EPIDOS, Epidemiology of Osteoporosis; F, females; M, males; MMSE, Mini Mental State Examination; OR, odds ratio; RR, relative risk; SPES, Swedish Performance Evaluating System; SPMSQ, Short Portable Mental Status Questionnaire; TMT, Trail Making test; yr, year.

## Data Availability

Data are contained within the article.
